# The relationship between substance use and self-reported aspects of social functioning in patients with a psychotic disorder

**DOI:** 10.1192/j.eurpsy.2024.9

**Published:** 2024-02-29

**Authors:** HS van der Heijden, Martijn Kikkert, Lieuwe de Haan, Menno Segeren, Simone Molman, Frederike Schirmbeck, Jentien Vermeulen

**Affiliations:** 1 Department of Psychiatry, Amstredam UMC, Location University of Amsterdam, Amsterdam, The Netherlands; 2 Department of Research, Arkin Mental Health Care, Amsterdam, The Netherlands; 3 Department of Healthy Living, Public Health Service Amsterdam, Amsterdam, The Netherlands; 4 Department of Public Mental Health, Central Institute of Mental Health, Medical Faculty Mannheim, Heidelberg University, Mannheim, Germany

**Keywords:** alcohol, cannabis, psychotic disorder, smoking, social functioning

## Abstract

**Background:**

In patients with a psychotic disorder, rates of substance use (tobacco, cannabis, and alcohol) are higher compared to the general population. However, little is known about associations between substance use and self-reported aspects of social functioning in patients with a psychotic disorder.

**Methods:**

In this cross-sectional study of 281 community-dwelling patients with a psychotic disorder, linear regression models were used to assess associations between substance use (tobacco, cannabis, or alcohol) and self-reported aspects of social functioning (perceived social support, stigmatization, social participation, or loneliness) adjusting for confounders (age, gender, and severity of psychopathology).

**Results:**

Compared to nonsmokers, both intermediate and heavy smokers reported lower scores on loneliness (E = −0.580, SE = 0.258, p = 0.025 and E = −0.547, SE = 0,272, p = 0.046, respectively). Daily cannabis users reported less social participation deficits than non-cannabis users (E = −0.348, SE = 0.145, p = 0.017). Problematic alcohol use was associated with more perceived social support compared to non-alcohol use (E = 3.152, SE = 1.102, p = 0.005). Polysubstance users reported less loneliness compared to no users (E = −0.569, SE = 0.287, p = 0.049).

**Conclusions:**

Substance use in patients with psychosis is associated with more favorable scores on various self-reported aspects of social functioning.

## Introduction

In patients with psychosis, disruptions in social functioning are a fundamental characteristic of the disease [1]. One of the factors that is studied in relation to social functioning is substance use [2]. The prevalence of substance use is extremely high among patients with psychosis [3]. To illustrate, a large cohort study [4] demonstrated that 62% of individuals diagnosed with a psychotic disorder reported current tobacco smoking, compared to 20% of the general population [5]. Cannabis use among patients with psychotic disorders is more than twice as high as that among the general population [6]. With respect to alcohol, patients with a psychotic disorder exhibit a fourfold increased risk of heavy alcohol consumption [7]. Importantly, the use of substances in psychotic disorders is associated with several adverse effects, including a threefold increase in psychotic relapses [8] and higher rates of violence and suicides compared to nonusers [9].

The association between substance use and social functioning remains less clear. In a meta-analysis conducted by Large et al. [2] among patients with psychosis, the overall conclusion was that substance use was *not* linked to diminished social functioning. It is noteworthy, however, that the term “social functioning” is a broad concept encompassing several components. In the aforementioned meta-analysis [2], social functioning in the majority of studies was defined by clinician-rated assessments. Some authors concluded that patients’ perspectives on aspects of social functioning in psychosis may differ from assessments made by clinicians [10]. There is an increasing effort to encompass the personal experience of participation and recovery in patients with psychosis [11]. In that light, studying the patient’ perspective on social functioning might be of interest.

When focusing on self-reported aspects of social functioning, loneliness was positively associated with comorbid drug or alcohol use [12]. Furthermore, one fairly recent study in patients with psychosis showed that smoking was negatively associated with self-reported participation in prosocial activities [13]. Another study in psychosis found that patients with problematic alcohol use reported more problems in interpersonal and family relationships [14]. To the best of our knowledge, the relationship between cannabis use and self-reported aspects of social functioning among patients with psychosis has not yet been studied.

Taken together, the literature on the relationships between substance use and various aspects of self-reported social functioning in patients with a psychotic disorder is scarce. In most studies that explored these relationships, clinician-rated scales [15–17] were used, thereby missing potential important self-reported information. What is more, the majority of studies concentrated on general substance use (including cannabis, alcohol, and stimulants), rather than exploring the relationships between social aspects and specific substances separately. This approach may overlook possible different associations between different substances and self-reported social aspects.

Therefore, the aim of the current study is to examine the specific associations of tobacco, cannabis or alcohol use with different self-reported aspects of social functioning (perceived social support, stigmatization, reported social participation, and loneliness).

## Method

### Study sample and design

A cross-sectional study was performed that was based on a local research cohort “Deinstitutionalization Amsterdam.” This cohort consisted of a representative sample of adults with a severe mental illness living independently (i.e., not clinically admitted). Participants were recruited from the SMI patient populations of the two largest mental health care institutions located in Amsterdam, where they receive mental health care. Detailed information about the composition of this cohort and the study procedures is described elsewhere [18]. In the current study, we included 281 patients diagnosed with a psychotic disorder (according to the DSM-V criteria). Within this sample, 172 (61.2%) patients had a schizophrenia spectrum disorder (DSM-IV 295.4 and 295.9), 56 (19.9%) were diagnosed with a schizoaffective disorder (DSM-IV 295.7), and 53 (18.9%) had a diagnosis of unspecified psychosis (293.81, 297.1, and 298.8). Inclusion criteria were an age range from 18 to 65 years and proficiency in either Dutch or English. The study was approved by the medical ethical review committee of the Amsterdam University Medical Centre (METC-AMC W16_276 # 16.324). Prior to the interview, participants provided written informed consent concerning both the interview and retrieval of information from patient files. All participants received a financial compensation of 15 euros. The data were obtained by research assistants from the mental health care institutions who interviewed the participants using a series of structured and validated questionnaires on among others substance use and social functioning. Data were collected between December 2017 and February 2020.

### Assessment instruments

#### Substance use

Severity and frequency of substance use (tobacco, cannabis, and alcohol) were assessed using the Measurements in the Addictions for Triage and Evaluation, which was found to be reliable in patients with substance use problems [19].

With respect to tobacco use, all patients were asked how much they smoked on average per day in the past 30 days: 0 cigarettes/day (nonusers), 1–9 cigarettes/day (low users), 10–19 cigarettes/day (moderate users), and 20 or more cigarettes/day (heavy users) [20]. In the current analyses, low and moderate users (1–19 cigarettes per day) were collapsed into a single user group due to the small sample sizes, which would limit the power of the analyses.

The use of cannabis was assessed by asking participants how many days they used cannabis on average in the past 30 days [21]. Patients were categorized into three different categories: nonusers, non-daily users, and daily users [22].

Hazardous alcohol use was assessed with the Alcohol Use Disorders Identification Test (AUDIT) [23], ranging from 0 to 40, which differentiates between non-problematic and problematic alcohol use. Cut-off cores were set at eight or higher in males and six or higher in females [24].

#### Self-reported aspects of social functioning

Four different self-reported aspects of social functioning were included in the current study: social support, stigmatization, social participation, and loneliness.

The Social Support List (SSL-12), a short version of the SSL interactions [25], was used to measure social support [26]. The instrument comprises 12 items that assess perceived social support by means of social interactions with members of one’s primary social network. For example, patients were asked if someone had ever shown interest in them. Each item has four response options ranging from 1 (seldom or never) to 4 (very often). Higher scores indicated a higher degree of perceived social support. Sum scores were used for the analyses. The internal reliability estimates for all assessments exceeded 0.70, supporting previous research findings indicating satisfactory psychometric properties of the SSL-12 [26].

Stigmatization was measured using the Stigma Scale [27], a 28-item self-report questionnaire with three subscales: disclosure (self-stigma), discrimination (experienced stigma), and potential positive aspects emanating from having a psychotic disorder. Patients were asked to report how much they (dis)agreed with several statements on a 5-point Likert scale ranging from 0 (strongly disagree) to 4 (strongly agree). The sum score per subscale was used for the analyses. Higher scores indicate more perceived stigmatization. To maintain consistency across all subscales, the scoring of the questionnaire was reserved for items that explored positive aspects of mental illness. The psychometric properties of the Stigma Scale have been previously found to be sufficient (Cronbach’s α 0.87) [27].

To determine social participation, the Social Exclusion Index (SEI-HS) was used [28]. This questionnaire consists of four different dimensions: “social participation,” “material deprivation,” “normative integration,” and “access to basic social rights.” In the current study, only the dimension “social participation” was included, as the main goal was to evaluate social participation. Patients were asked to report on four different statements (e.g., there are people with whom I can have a good conversation) on a 3-point Likert scale ranging 1–3 (yes, sometimes, and no). Higher scores indicated poorer social participation (i.e., more lack of social participation). A mean score of the four items was calculated after adjustment of the raw scores and subsequently applying weighted item scores, according to the guidelines of the instrument [28]. Internal consistency for the subscale “social participation” was sufficient (Cronbach’s α 0.75). The authors [29] assessed the construct validity of the SEI-HS by testing specific hypotheses concerning the relationship between the SEI-HS scores and various risk factors and considered it adequate [30].

Loneliness was measured using the short version of the Loneliness Scale [31]. Participants responded to six statements (e.g., I miss having people around me) on a 5-point scale, ranging from 1 (strongly disagree) to 5 (strongly agree). Higher scores indicated more loneliness. A mean score was calculated for the analyses after recoding the negatively formulated item and dichotomizing all items. The short Loneliness Scale has been shown to be a valid and reliable instrument for measuring loneliness. Internal consistency was previously found to be satisfactory (Cronbach’s α ≥ 0.70) [32].

### Covariates

All participants provided information on sociodemographic features. A priori, we selected age, gender, and severity of psychopathology as potential confounders as these variables are associated with substance use and several aspects of social functioning [33, 34]. To rate the severity of psychopathology, the Brief Psychiatric Rating Scale (BPRS) was administered [35]. The BPRS is a widely used 24-item tool to measure the presence and severity of several psychiatric symptoms and behavior in patients with a psychiatric disorder by observation and self-reports during a semi-structured interview.

The BPRS consists of five different subscales (positive symptoms, negative symptoms, affectivity, resistance, and activation). In the analyses, the BPRS total score was included as a covariate. The BPRS has been tested for validity and reliability, which appeared to be satisfactory [36].

### Statistical analyses

Statistical analyses were performed using IBM SPSS Statistics version 26. The normality of the distribution of scores on the outcomes of interest was assessed by visual inspection of the histograms, which showed no deviations from normality. Linear regression models were used to evaluate the associations between substance use and social aspects. Each substance was entered as an independent variable with each of the self-reported aspects of social functioning as the dependent variable while correcting for age, gender, and psychopathology. As the independent variables were multi-categorical, dummy variables were created with “no use” being the reference group. As sensitivity analyses, a second set of linear regression analyses, were performed with the independent variables number of cigarettes smoked per day and the amount of cannabis smoked per day (uniformed in grams) instead of the multi-categorical variables. Finally, we explored the potential differences in self-reported aspects of social functioning between no users, single substance users, and polysubstance users (≥two substances). All statistical tests were two-sided, and significance was set at p < 0.05.

## Results

As shown in Table 1, all participants had data available on tobacco, cannabis use, and alcohol use. Summary scores of the self-reported aspects of social functioning per substance group are listed in Supplement 1.

**Table 1.** Demographic and clinical characteristics of patients with a psychotic disorder (N = 281)

Gender	
Male	193 (68.7%)
Female	88 (31.3%)
Age (years)	48 (9.8)
Education^a^	N = 279
Primary education	181 (64.9%)
Secondary education	69 (24.7%)
Higher education	29 (10.4%)
Psychopathology	
BPRS – positive symptoms	1.93 (1.03), N = 274
BPRS – negative symptoms	1.72 (0.74), N = 273
BPRS – depressive symptoms	2.03 (0.90), N = 272
BPRS – disorganization symptoms	1.43 (0.47), N = 273
BPRS – mean total score	1.75 (0.52), N = 273
Tobacco	
No use (0 cigarette/day)	123 (43.8%)
Low-intermediate use (1–19 cig/day)	82 (29.2%)
Heavy use (≥20 cigarettes/day)	76 (27.0%)
Cannabis use	
Non-use	216 (76.9%)
Non-daily use	38 (13.5%)
Daily use	27 (9.6%)
Alcohol use	
No use	106 (37.7%)
Non-problematic use	112 (39.9%)
Problematic use	63 (22.4%)
Polysubstance use^b^	
No substances	59 (21.0%)
Single substance	92 (32.7%)
Two substances	78 (27.8%)
Three substances	52 (18.5%)

*Note*: Data are N (%) or mean (SD).

Abbreviations: BPRS, brief psychiatric rating scale.

a Categories are defined as follows: primary education including prevocational education, secondary education including pre-university education, and higher education including higher vocational and academic education according to the Dutch educational system (Rauschenberg et al., 2021).

b Including tobacco, cannabis, and/ or alcohol.

The prevalence estimate of tobacco use was 55.8%, and tobacco users smoked 19 cigarettes per day on average. A total of 23.1% patients used cannabis, with 13.5% on a non-daily and 9.6% on a daily basis. With respect to alcohol, 62.3% of all participants used alcohol and 22.4% were problematic alcohol users. Concerning polysubstance use, 92 (32.7%) patients used one substance (tobacco, cannabis, or alcohol) and 130 (46.3%) patients were polysubstance users, of which 78 (27.8%) used two of the above-mentioned substances and 52 (18.5%) used all three substances.

### Tobacco

Compared to nonsmokers, both intermediate and heavy smoking patients showed lower scores on loneliness (E = −0.580, SE = 0.258, p = 0.025 and E = −0.547, SE = 0,272, p = 0.046, respectively) when correcting for confounders, as shown in Table 2. A post hoc analysis (Supplement 2) revealed a negative association between the number of cigarettes smoked per day and levels of loneliness (E = −0.017, SE = 0.008, p = 0.042). Perceived social support, reported social participation, and stigmatization were not significantly associated with tobacco after adjusting for confounders.

**Table 2.** Results of regression models evaluating the associations between substances and self-reported aspects of social functioning (N = 281)

	Intermediate smoker versus No smoker	Heavy smoker versus No smoker
*Outcome variable*	Estimate (SE) [95% CI], p-value	Estimate (SE) [95% CI], p-value
Social support	0.053 (0.992) [−1.900–2.005], p = 0.958	0.858 (1.052) [−1.214–2.930], p = 0.416
Stigmatization – disclosure	−1.237 (1.186) [−0.079 – −1.043], p = 0.298	−0.631 (1.288) [−0.028 – −0.490], p = 0.625
Stigmatization – discrimination	−1.028 (1.138) [−3.274–1.217], p = 0.367	2.055 (1.248) [−0.406–4.516], p = 0.101
Stigmatization – positive aspects	0.706 (0.495) [−0.269–1.681], p = 0.155	0.979 (0.533) [−0.072–2.031], p = 0.068
Lack of social participation	0.035 (0.100) [−0.163–0.232], p = 0.729	0.075 (0.107) [−0.134–0.285], p = 0.479
Loneliness	−0.580 (0.258) [−1.088 – −0.072], p = 0.025*	−0.547 (0.272) [−1.083 – −0.011], p = 0.046*
	*Non-daily cannabis versus No cannabis*	*Daily cannabis versus No cannabis*
*Outcome variable*	Estimate (SE) [95% CI], p-value	Estimate (SE) [95% CI], p-value
Social support	1.475 (1.237) [−0.960–3.910], p = 0.234	0.567 (1.444) [−2.276–3.411], p = 0.695
Stigmatization – disclosure	−0.984 (1.500) [−3942 – −1.974], p = 0.513	0.671 (1.663) [−2.609–3.950], p = 0.687
Stigmatization – discrimination	0.792 (1.431) [−2.030–3.615], p = 0.580	−0.623 (1.638) [−3.854–2.607], p = 0.704
Stigmatization – positive aspects	0.524 (0.625) [−0.709–1.757], p = 0.403	0.351 (0.715) [−1.058–1.760], p = 0.624
Lack of social participation	−0.115 (0.124) [−0.359–0.129], p = 0.355	−0.348 (0.145) [−0.633 – −0.063], p = 0.017*
Loneliness	−0.203 (0.324) [−0.804–0.434], p = 0.531	0.276 (0.382) [−0.475–1.028], p = 0.470
	*Non-problematic alcohol versus No alcohol*	*Problematic alcohol versus No alcohol*
*Outcome variable*	Estimate (SE) [95% CI], p-value	Estimate (SE) [95% CI], p-value
Social support	0.883 (0.912) [−0.912–2.678], p = 0.334	3.152 (1.102) [0.982–5.321], p = 0.005*
Stigmatization – disclosure	0.193 (1.123) [−2.021–2.408], p = 0.864	−1.618 (1.372) [−4.323–1.086], p = 0.239
Stigmatization – discrimination	−0.148 (1.085) [−2.288–1.993], p = 0.892	1.175 (1.321) [−1.431–3.781], p = 0.375
Stigmatization – positive aspects	0.700 (0.466) [−0.219–1.619], p = 0.135	0.222 (0.572) [−0.906–1.350], p = 0.698
Lack of social participation	−0.044 (0.093) [−0.227–0.139], p = 0.637	−0.200 (0.113) [−0.422–0.022], p = 0.078
Loneliness	−0.077 (0.243) [−0.554–0.401], p = 0.544	−0.350 (0.292) [−0.926–0.225], p = 0.232

*Note*: Estimate (standard error). [95% CI] = 95% confidence interval. Higher scores indicate poorer outcomes, with the exception of the social support outcome. No use is the reference category against which other levels of use are compared. All estimates are corrected for age, gender, and psychopathology.

* *p* <0.05

### Cannabis

Patients who used cannabis daily reported less social participation deficits than patients who did not use cannabis (E = −0.348, SE = 0.145, p = 0.017). In a sensitivity analysis, we explored whether a dose–response relationship existed between the amount of cannabis smoked per day (uniformed in grams) and reported social participation. No significant association was found, as shown in Supplement 3. Neither perceived social support, reported stigmatization, nor loneliness were significantly associated with cannabis use when correcting for confounders (Table 2).

### Alcohol use

Problematic alcohol use was associated with more perceived social support compared to no-alcohol use (E = 3.152, SE = 1.102, p = 0.005) after adjusting for confounders, as shown in Table 2. Given differences in the literature regarding the cut-off scores for problematic alcohol use [24, 37], we also explored whether this association remained significant when raising the AUDIT cut-off score to 16, indicating severe problematic use [37]. As shown in Supplement 4, outcomes were not affected (E = 3.394, SE = 1.581, p = 0.033). Alcohol use was not significantly related to social participation, stigmatization, and loneliness when correcting for all confounders.

### Polysubstance use

Patients with polysubstance use reported less loneliness compared to no users (E = −0.569, SE = 0.287, p = 0.049) (Supplement 5). No significant differences were found between no users, single substance users, and polysubstance users on all other self-reported aspects of social functioning.

## Discussion

### Main findings

The current study mapped substance use among community-dwelling patients with a psychotic disorder in the Netherlands and examined associations between the use of specific substances (tobacco, cannabis, alcohol) and several self-reported aspects of social functioning.

Tobacco use (intermediate and heavy use) was associated with less self-reported loneliness compared to those who did not smoke. Patients with cannabis use reported more social participation compared to non-cannabis-using patients. Problematic alcohol use was associated with more self-reported social support compared to non-problematic alcohol use. On the subscales of the stigma scale (including self-stigma [disclosure] and experienced stigma [discrimination]), no differences were found between any of the substance user groups. Finally, we found that self-reported loneliness was less prevalent among patients with polysubstance use than among nonusers. In summary, severe use of both cannabis and alcohol seems to be associated with better self-reported scores on some of the outcomes investigated. Concerning tobacco, this relationship was also found in patients with low to intermediate use and heavy use.

### Current findings in the light of previous literature

The prevalence estimate of tobacco use in our study (55.8%) is consistent with the rate (62%) found in a large population of patients with a psychotic disorder [4]. With respect to cannabis, the prevalence of daily use in our study population (9.6%) was lower compared to rates of cannabis abuse in psychotic patients in general (20%), as shown in a meta-analysis [38]. Concerning alcohol, the prevalence estimate of problematic use of 22.4% obtained in our study was consistent with the literature [39]. With respect to polysubstance use, our prevalence rate of 46.3% was higher compared to rates (30%) found by another study [40]. This could be explained by the fact that the latter study only included patients who *misused* two or more substances, whereas in our study, patients who *used* two or more substances were also included. One study [41] suggested that cannabis use can be regarded as a proxy-measure for the use of other drugs. This notion appears to be confirmed in our study, as there were only two patients who used cannabis but none of the other substances.

To the best of our knowledge, no previous studies evaluated the relationship between tobacco use (smoking) and perceived loneliness in patients with a psychotic disorder. Smoking was associated with less perceived loneliness. The direction of this association remains unclear. Based on findings in the general population, where three large studies [42–44] concluded that smoking *increased* self-reported feelings of loneliness and social isolation, this finding was contrary to our expectations.

One could argue that assessments made by clinicians may differ from those self-reported by patients with psychosis. Indeed, a study indicated that elevated levels of perceived loneliness in individuals with psychosis were not dependent on objective social isolation [12], challenging the assumption that perceived loneliness is solely a consequence of limited social contact in psychosis. In that light, it is of interest to explore in future psychosis studies the relationship between substance use and both self-reported and clinician-rated aspects of social functioning. Furthermore, it is widely recognized that patients with psychosis experience increased feelings of loneliness compared to the general population [45]. For patients experiencing psychosis, smoking was perceived as a way to find relief from loneliness [46, 47] – even though this may not be associated with a reduction in objective social isolation.

To the best of our knowledge, no other studies evaluated the relationship between cannabis and self-reported social participation. The existing literature utilizing clinician-rated scales on social aspects reported conflicting findings. For instance, a study [48] demonstrated that patients without cannabis use disorder showed significant improvements in social functioning over time. This aligns with two other studies reporting that continued cannabis correlated with poorer social functioning [49–51], while others concluded that cannabis use was linked to enhanced global social functioning in individuals with an increased risk for psychosis [52]. Given these conflicting results, future studies could investigate the relationship between cannabis use and both self-reported and clinician-rated aspects of social functioning.

An explanation for the positive relationship between cannabis and more self-reported social participation in our study could also be related to potential differences in the patients’ profile: it might be that cannabis use may precipitate psychosis in individuals who would otherwise not transit to a psychotic disorder [53], compared to patients who converted without using cannabis [54]. This explanation is in line with the vulnerability-stress model, in which cannabis use is a risk factor to pass the threshold and develop a psychotic disorder [55]. Hence, those with cannabis use and a psychotic disorder might be better socially functioning with less severe cognitive impairments than non-cannabis-using patients, who are thought to be more vulnerable due to genetic factors [56]. Another explanation is that cannabis use occurs within a bidirectional social-cannabis context, wherein the craving for cannabis adds active engagement with a specific peer group mutually reinforced each other, resulting in increased cannabis consumption.

With respect to alcohol, we found that problematic alcohol use was associated with higher scores on perceived social support, which was at odds with another study [14], where patients reported more alcohol associated-problems in interpersonal and family relationships compared to non-problematic users. This is in line with Cetty et al [57], who found that hazardous alcohol use predicted lower self-reported scores on social relationships in patients with a first-episode psychosis. Differences in the exact outcome scores (perceived social support vs. the quality of relationships) might play a role in these divergent findings.

It might be that patients with increased social interaction are more at risk for problematic alcohol use as they may go along with individuals with problematic alcohol use. Some support for this explanation comes from a recent study conducted in the general population that found that adolescents with less social interest used alcohol less frequently [58]. Furthermore, a number of psychosis studies have found that alcohol use, including binge drinking [59] and hazardous drinking [57], was associated with *lower* severity of negative symptoms [60–62]. It has been suggested that impaired rewarding systems in patients with severe negative symptoms could be associated with diminished reinforcing effects of alcohol use [61]. Given the overlap between lower negative symptom severity and higher social support as an outcome in the current study, this could play a role in explaining our findings.

We did not find any significant relationship between any of the substances and perceived stigmatization. It is, nonetheless, important to acknowledge that in one study, patients reported to smoke to relief feelings of stigma by trying to “fit in” [46]. Correspondingly, smoking *cessation* was associated with fear of being socially ostracized when quitting smoking [63]. However, in the past decades, smoking actually became *more* associated with stigma [64]. Furthermore, previous literature found that especially those who experience disapproval by others over their inability to give up tobacco feel stigmatized [64]. Patients with a psychotic disorder can be seen as harder-to-treat smokers [65]. Therefore, efforts should be directed toward assisting patients in smoking cessation, not least because quitting has also been associated with reductions in public stigma and discrimination [66] along with decreases of depression, anxiety, and stress [67].

### Strengths and limitations

The strengths of the current study are the relatively large sample, representative of patients with psychotic disorders living in the community, and the evaluation of different substances on several self-reported aspects of social functioning, allowing for an in-depth examination of separate relationships between substance use and self-reported aspects of social functioning in patients with a psychotic disorder. However, the current study has several limitations. First, the cross-sectional design of the current study lacks the capability of evaluating the potential causal effects of substance use behavior in relation to several self-reported aspects of social functioning. Second, reverse causality and residual confounding cannot be ruled out due to the cross-sectional analyses and observational design. Third, information concerning the use of medication was missing. Fourth, given the explorative design of the current study, we chose to set the statistical significance at p < 0.05 instead of using a more conservative approach as Bonferroni correction for multiple testing. However, by this decision, we introduced the risk of a type-I error. Fifth, the majority of the study population was male (68.7%) and lower educated (64.9%), although these characteristics are probably representative for the group of patients with longstanding psychotic disorders. Sixth, the assessment of frequency and severity of substance use was based on self-report. Hence, under- or overestimation of substance use cannot be ruled out. Seventh, we did not correct for polysubstance use in our main analyses as dissecting out individual substance use would have drastically decreased the sample size. Nevertheless, we aimed to explore the effects of comorbid use by analyzing no users, single users, and polysubstance users. Finally, the outcome “social participation” was based on one subscale of the SEI-HS that included only four items. This could potentially have led to low content validity.

### Clinical implications

In the current study, smoking was associated with less perceived loneliness. As the negative health consequences of tobacco are disastrous, patients should be encouraged to stop smoking. As smoking may be a maladaptive coping strategy, it is beneficial to the patient to replace smoking with healthier coping skills. Referring the patient for psychological and behavioral treatment is required.

Therapists should be alert on the possibility that loneliness may be an issue and that individuals who try to stop smoking may need support in this respect. In the Netherlands, referral to a certified coach for psychological smoking cessation interventions is covered by the health insurance and should be offered more consequently to patients with psychosis.

In the current sample, psychotic patients who use cannabis reported betters scores on social participation. Consequently, for some patients, it might be that cannabis use is considered helpful in facilitating social interactions. However, the overwhelming (long-term) negative effects of cannabis have been documented [68], and cannabis reduction had favorable effects on symptoms and social functioning [69]. Therefore, it is important to help patients in finding education, work, or other structured activities, as these are crucial elements for enabling a reduction in cannabis use and, at the same time, finding alternative social participation [70, 71].

Concerning current findings of the association between (heavy) alcohol use and self-reported aspects of social functioning in patients with a psychotic disorder, longitudinal studies are needed to explore causality.

In the current study, polysubstance users reported less loneliness compared to patients who did not use drugs. A review that explored the reasons for polysubstance use [72] concluded that substances are generally combined to improve experiences and to increase/prolong a state of euphoria. It might be that negative feelings of loneliness are masked by a “high” state caused by polysubstance use. Nonetheless, psychosis risk was found to be associated with the cumulative effect of polysubstance use [41]. What is more, polysubstance use is also related to more psychiatric and psychosocial problems [73]. In future research, it might be interesting to explore other social aspects (for example functional or symptomatic recovery) in polysubstance users compared to single users and no users.

## Supporting information

van der Heijden et al. supplementary material van der Heijden et al. supplementary material

## Supplementary material

The supplementary material for this article can be found at http://doi.org/10.1192/j.eurpsy.2024.9.

## References

[1] Metzak PD, Farris MS, Placsko T, Braun A, Bonneville D, Brummitt K, et al. Social functioning and brain imaging in individuals at clinical high-risk for psychosis: A systematic review. Schizophr Res. 2021;233:3–12. 34126554 10.1016/j.schres.2021.04.013 PMC8380704

[2] Large M, Mullin K, Gupta P, Harris A, Nielssen O. Systematic meta-analysis of outcomes associated with psychosis and co-morbid substance use. Aust N Z J Psychiatry. 2014;48(5):418–32. 24589980 10.1177/0004867414525838

[3] Lähteenvuo M, Batalla A, Luykx JJ, Mittendorfer-Rutz E, Tanskanen A, Tiihonen J, et al. Morbidity and mortality in schizophrenia with comorbid substance use disorders. Acta Psychiatr Scand. 2021;144:42–9. 33650123 10.1111/acps.13291 PMC8359349

[4] Dickerson F, Schroeder J, Katsafanas E, Khushalani S, Origoni AE, Savage C, et al. Cigarette smoking by patients with serious mental illness, 1999–2016: An increasing disparity. Psychiatr Serv. 2018;69(2):147–53. 28945183 10.1176/appi.ps.201700118

[5] WHO. WHO global report on trends in prevalence of tobacco use 2000–2025, third edition. Geneva: World Health Organization; 2020.

[6] Arseneault L, Cannon M, Witton J, Murray RM. Causal association between cannabis and psychosis: Examination of the evidence. Br J Psychiatry. 2004;184(2):110–7. 14754822 10.1192/bjp.184.2.110

[7] Hartz SM, Pato CN, Medeiros H, Cavazos-Rehg P, Sobell JL, Knowles JA, et al. Comorbidity of severe psychotic disorders with measures of substance use. JAMA Psychiatry. 2014;71(3):248–54. 24382686 10.1001/jamapsychiatry.2013.3726 PMC4060740

[8] Alvarez-Jimenez M, Priede A, Hetrick SE, Bendall S, Killackey E, Parker AG, et al. Risk factors for relapse following treatment for first episode psychosis: A systematic review and meta-analysis of longitudinal studies. Schizophr Res. 2012;139(1):116–28. 22658527 10.1016/j.schres.2012.05.007

[9] Darke S, Lappin J, Farrell M. The clinician’s guide to illicit drugs and health. United Kingdom: Silverback Publishing; 2019.

[10] Jongs N, Penninx B, Arango C, Ayuso-Mateos JL, van der Wee N, Winter-van Rossum I, et al. Effect of disease related biases on the subjective assessment of social functioning in Alzheimer’s disease and schizophrenia patients. J Psychiatr Res. 2022;145:302–8. 33221026 10.1016/j.jpsychires.2020.11.013

[11] Simonsen C, Faerden A, Ueland T, Vaskinn A, Bjella T, Andreassen O, et al. Self-rated disability in first treated episode of psychosis: A 1-year follow-up study. Compr Psychiatry. 2018;85:48–54. 29981504 10.1016/j.comppsych.2018.06.004

[12] Trémeau F, Antonius D, Malaspina D, Goff DC, Javitt DC. Loneliness in schizophrenia and its possible correlates. An exploratory study. Psychiatry Res. 2016;246:211–7. 27721059 10.1016/j.psychres.2016.09.043

[13] Dekker TEG, van der Heijden HS, Schirmbeck F, van Amelsvoort T, Bartels-Velthuis AA, Simons CJP, et al. The association between smoking behaviour, social cognition and social functioning in patients with a non-affective psychotic disorder: A prospective follow-up study. Schizophr Res Cogn. 2021;26:100206. 34258239 10.1016/j.scog.2021.100206 PMC8259295

[14] Salyers MP, Mueser KT. Social functioning, psychopathology, and medication side effects in relation to substance use and abuse in schizophrenia. Schizophr Res 2001;48(1):109–23. 11278158 10.1016/s0920-9964(00)00063-3

[15] Cantwell R. Substance use and schizophrenia: Effects on symptoms, social functioning and service use. Br J Psychiatry. 2003;182:324–9. 12668408 10.1192/bjp.182.4.324

[16] Drake RE, Brunette MF, Mueser KT. Substance use disorders and social functioning in schizophrenia. In K. T. Mueser & N. Tarrier (Eds.) Handbook of social functioning in schizophrenia, Boston, MA: Allyn & Bacon; 1998, p. 280–9. https://www.researchgate.net/publication/313520393_Substance_use_disorders_and_social_functioning_in_schizophrenia

[17] Swartz MS, Wagner HR, Swanson JW, Stroup TS, McEvoy JP, McGee M, et al. Substance use and psychosocial functioning in schizophrenia among new enrollees in the NIMH CATIE study. Psychiatr Serv. 2006;57(8):1110–6. 16870961 10.1176/ps.2006.57.8.1110

[18] Segeren M, Lauriks S, Kikkert M, Heering J, Lommerse N, van Husen G, et al. Deinstitutionalization from the perspective of community-dwelling adults with a severe mental illness in Amsterdam: A cohort study protocol. BMC Public Health. 2022;22(1):950. 35549681 10.1186/s12889-022-13291-w PMC9097409

[19] Schippers GM, Broekman TG, Buchholz A, Koeter MW, van den Brink W. Measurements in the addictions for triage and evaluation (MATE): An instrument based on the World Health Organization family of international classifications. Addiction. 2010;105(5):862–71. 20331557 10.1111/j.1360-0443.2009.02889.x

[20] Ward HB, Lawson MT, Addington J, Bearden CE, Cadenhead KS, Cannon TD, et al. Tobacco use and psychosis risk in persons at clinical high risk. Early Interv Psychiatry. 2019;13(5):1173–81. 30362261 10.1111/eip.12751 PMC11531344

[21] Coutinho LS, Honorato H, Higuchi CH, Cavalcante DA, Belangeiro S, Noto M, et al. Cannabis acute use impacts symptoms and functionality in a cohort of antipsychotic naive first episode of psychosis individuals. Schizophr Res Cogn. 2019;16:12–6. 30581766 10.1016/j.scog.2018.10.002 PMC6293028

[22] Di Forti M, Sallis H, Allegri F, Trotta A, Ferraro L, Stilo SA, et al. Daily use, especially of high-potency cannabis, drives the earlier onset of psychosis in cannabis users. Schizophr Bull. 2014;40(6):1509–17. 24345517 10.1093/schbul/sbt181 PMC4193693

[23] Babor T, Higgins-Biddle JC, Saunders JB, Monteiro MG. AUDIT: The alcohol use disorders identification test: Guidelines for use in primary health care. 2nd ed. Geneva: World Health Organization; 2001.

[24] Nehlin C, Fredriksson A, Jansson L. Brief alcohol screening in a clinical psychiatric population: Special attention needed. Drug Alcohol Rev. 2012;31(4):538–43. 21726312 10.1111/j.1465-3362.2011.00333.x

[25] van Sonderen FLP. Het meten van sociale steun. Ph.D. Thesis; 1991.

[26] Kempen G, Eijk L. The psychometric properties of the SSL12-I, a short scale for measuring social support in the elderly. Soc Indic Res. 1995;35:303–12.

[27] King M, Dinos S, Shaw J, Watson R, Stevens S, Passetti F, et al. The stigma scale: Development of a standardised measure of the stigma of mental illness. Br J Psychiatry. 2007;190:248–54. 17329746 10.1192/bjp.bp.106.024638

[28] van Bergen APL, Hoff SJM, van Ameijden EJC, van Hemert AM. Measuring social exclusion in routine public health surveys: Construction of a multidimensional instrument. PLoS One. 2014;9(5):e98680. 24878842 10.1371/journal.pone.0098680 PMC4039524

[29] van Bergen APL, Hoff SJM, Schreurs H, van Loon A, van Hemert AM. Social exclusion index-for health surveys (SEI-HS): A prospective nationwide study to extend and validate a multidimensional social exclusion questionnaire. BMC Public Health. 2017;17(1):253. 28288609 10.1186/s12889-017-4175-1 PMC5348771

[30] Terwee CB, Bot SDM, de Boer MR, van der Windt DAWM, Knol DL, Dekker J, et al. Quality criteria were proposed for measurement properties of health status questionnaires. J Clin Epidemiol. 2007;60(1):34–42. 17161752 10.1016/j.jclinepi.2006.03.012

[31] de Jong-Gierveld J, van Tilburg TG. Manual of the Loneliness Scale. Methoden en technieken; 1999, 10.17605/osf.io/u6gck.

[32] de Jong-Gierveld J, van Tilburg T. [A shortened scale for overall, emotional and social loneliness]. Tijdschr Gerontol Geriatr. 2008;39(1):4–15. 18365511 10.1007/BF03078118

[33] Weibell MA, Hegelstad WtV, Auestad B, Bramness J, Evensen J, Haahr U, et al. The effect of substance use on 10-year outcome in first-episode psychosis. Schizophr Bull. 2017;43(4):843–51. 28199703 10.1093/schbul/sbw179 PMC5472130

[34] de Winter L, Couwenbergh C, van Weeghel J, Hasson-Ohayon I, Vermeulen JM, Mulder CL, et al. Changes in social functioning over the course of psychotic disorders: A meta-analysis. Schizophr Res. 2022;239:55–82. 34844096 10.1016/j.schres.2021.11.010

[35] Overall JE, Gorham DR. The brief psychiatric rating scale. Psychol Rep. 1962;10(3):799–812.

[36] Thomas A, Donnell AJ, Young TR. Factor structure and differential validity of the expanded brief psychiatric rating scale. Assessment 2004;11(2):177–87. 15171466 10.1177/1073191103262893

[37] Kumar CN, Thirthalli J, Suresha KK, Arunachala U, Gangadhar BN. Alcohol use disorders in patients with schizophrenia: Comparative study with general population controls. Addict Behav. 2015;45:22–5. 25634440 10.1016/j.addbeh.2015.01.009

[38] Koskinen J, Lohonen J, Koponen H, Isohanni M, Miettunen J. Rate of cannabis use disorders in clinical samples of patients with schizophrenia: A meta-analysis. Schizophr Bull. 2010;36(6):1115–30. 19386576 10.1093/schbul/sbp031 PMC2963055

[39] Hunt GE, Large MM, Cleary M, Lai HMX, Saunders JB. Prevalence of comorbid substance use in schizophrenia spectrum disorders in community and clinical settings, 1990–2017: Systematic review and meta-analysis. Drug Alcohol Depend. 2018;191:234–58. 30153606 10.1016/j.drugalcdep.2018.07.011

[40] Wade D, Harrigan S, Edwards J, Burgess PM, Whelan G, McGorry PD. Substance misuse in first-episode psychosis: 15-month prospective follow-up study. Br J Psychiatry. 2006;189:229–34. 16946357 10.1192/bjp.bp.105.017236

[41] Shevlin M, McElroy E, Murphy J, Hyland P, Vallieres F, Elklit A, Christoffersen M. Cannabis and psychosis: The impact of polydrug use. Drugs Alcohol Today. 2017;17(3):186–94.

[42] Wootton RE, Greenstone HSR, Abdellaoui A, Denys D, Verweij KJH, Munafò MR, Treur JL. Bidirectional effects between loneliness, smoking and alcohol use: Evidence from a Mendelian randomization study. Addiction. 2021;116(2):400–6. 32542815 10.1111/add.15142

[43] Philip KE, Bu F, Polkey MI, Brown J, Steptoe A, Hopkinson NS, et al. Relationship of smoking with current and future social isolation and loneliness: 12-year follow-up of older adults in England. Lancet Reg Health Eur. 2022;14:100302. 35036984 10.1016/j.lanepe.2021.100302 PMC8743222

[44] Matsuyama Y, Tabuchi T. Does tobacco smoking increase social isolation? A Mendelian randomization study. Am J Epidemiol. 2023. doi:10.1093/aje/kwad229. PMC10999643 37981720

[45] Michalska da Rocha B, Rhodes S, Vasilopoulou E, Hutton P. Loneliness in psychosis: A meta-analytical review. Schizophr Bull. 2018;44(1):114–25. 28369646 10.1093/schbul/sbx036 PMC5768045

[46] Trainor K, Leavey G. Barriers and facilitators to smoking cessation among people with severe mental illness: A critical appraisal of qualitative studies. Nicotine Tob Res. 2017;19(1):14–23. 27613905 10.1093/ntr/ntw183

[47] Gregg L, Barrowclough C, Haddock G. Reasons for increased substance use in psychosis. Clin Psychol Rev. 2007;27(4):494–510. 17240501 10.1016/j.cpr.2006.09.004

[48] Gonzalez-Blanch C, Gleeson JF, Koval P, Cotton SM, McGorry PD, Alvarez-Jimenez M. Social functioning trajectories of young first-episode psychosis patients with and without cannabis misuse: A 30-month follow-up study. PLoS One. 2015;10(4):e0122404. 25849623 10.1371/journal.pone.0122404 PMC4388449

[49] Bruins J, Pijnenborg GHM, Visser E, Castelein S. The association of cannabis use with quality of life and psychosocial functioning in psychosis. Schizophr Res. 2021;228:229–34. 33461022 10.1016/j.schres.2020.11.059

[50] Faber G, Smid HG, Van Gool AR, Wunderink L, van den Bosch RJ, Wiersma D. Continued cannabis use and outcome in first-episode psychosis: Data from a randomized, open-label, controlled trial. J Clin Psychiatry. 2012;73(5):632–8. 22394457 10.4088/JCP.11m07069

[51] Seddon JL, Birchwood M, Copello A, Everard L, Jones PB, Fowler D, et al. Cannabis use is associated with increased psychotic symptoms and poorer psychosocial functioning in first-episode psychosis: A report from the UK national EDEN study. Schizophr Bull. 2015;42(3):619–25. 26536902 10.1093/schbul/sbv154 PMC4838086

[52] Auther AM, McLaughlin D, Carrión RE, Nagachandran P, Correll CU, Cornblatt BA. Prospective study of cannabis use in adolescents at clinical high risk for psychosis: Impact on conversion to psychosis and functional outcome. Psychol Med. 2012;42(12):2485–97. 22716931 10.1017/S0033291712000803 PMC3459073

[53] Peralta V, García de Jalón E, Moreno-Izco L, Peralta D, Janda L, Sánchez-Torres AM, Cuesta MJ. Long-term outcomes of first-admission psychosis: A naturalistic 21-year follow-up study of symptomatic, functional and personal recovery and their baseline predictors. Schizophr Bull. 2022;48(3):631–42. 34999894 10.1093/schbul/sbab145 PMC9077430

[54] Yücel M, Bora E, Lubman DI, Solowij N, Brewer WJ, Cotton SM, et al. The impact of cannabis use on cognitive functioning in patients with schizophrenia: A meta-analysis of existing findings and new data in a first-episode sample. Schizophr Bull. 2012;38(2):316–30. 20660494 10.1093/schbul/sbq079 PMC3283159

[55] Lemvigh C, Brouwer R, Hilker R, Anhøj S, Baandrup L, Pantelis C, et al. The relative and interactive impact of multiple risk factors in schizophrenia spectrum disorders: A combined register-based and clinical twin study. Psychol Med. 2021;53:1266–1276. 35822354 10.1017/S0033291721002749

[56] Kayir H, Ruffolo J, McCunn P, Khokhar JY. The relationship between cannabis, cognition, and schizophrenia: It’s complicated. Curr Top Behav Neurosci. 2023;63:437–61. 36318403 10.1007/7854_2022_396

[57] Cetty L, Shahwan S, Satghare P, Devi F, Chua BY, Verma S, et al. Hazardous alcohol use in a sample of first episode psychosis patients in Singapore. BMC Psychiatry. 2019;19(1):91. 30876474 10.1186/s12888-019-2073-z PMC6419799

[58] Pijnenburg LJ, Kaplun A, de Haan L, Janecka M, Smith L, Reichenberg A, et al. Autistic traits and alcohol use in adolescents within the general population. Eur Child Adolesc Psychiatry. 2023;32:1633–42. 35318541 10.1007/s00787-022-01970-3 PMC10460309

[59] Tan JH, Shahwan S, Satghare P, Cetty L, Verma S, Sendren JR, et al. Binge drinking: Prevalence, correlates, and expectancies of alcohol use among individuals with first-episode psychosis. Early Interv Psychiatry. 2019;13(5):1136–45. 30345621 10.1111/eip.12744 PMC6899451

[60] Archie S, Gyomorey K. First episode psychosis, substance abuse and prognosis: A systematic review. Curr Psychiatr Rev. 2009;5:153–63.

[61] Batki SL, Leontieva L, Dimmock JA, Ploutz-Snyder R. Negative symptoms are associated with less alcohol use, craving, and “high” in alcohol dependent patients with schizophrenia. Schizophr Res. 2008;105(1–3):201–7. 18701256 10.1016/j.schres.2008.06.020 PMC2582942

[62] Van Mastrigt S, Addington J, Addington D. Substance misuse at presentation to an early psychosis program. Soc Psychiatry Psychiatr Epidemiol. 2004;39(1):69–72. 15022049 10.1007/s00127-004-0713-0

[63] Filia SL, Baker AL, Gurvich CT, Richmond R, Kulkarni J. The perceived risks and benefits of quitting in smokers diagnosed with severe mental illness participating in a smoking cessation intervention: Gender differences and comparison to smokers without mental illness. Drug Alcohol Rev. 2014;33(1):78–85. 24256336 10.1111/dar.12091

[64] Castaldelli-Maia JM, Ventriglio A, Bhugra D. Tobacco smoking: From ‘glamour’ to ‘stigma’. A comprehensive review. Psychiatry Clin Neurosci. 2016;70(1):24–33. 26449875 10.1111/pcn.12365

[65] Zeng LN, Zong QQ, Zhang L, Feng Y, Ng CH, Ungvari GS, et al. Worldwide prevalence of smoking cessation in schizophrenia patients: A meta-analysis of comparative and observational studies. Asian J Psychiatr. 2020;54:102190. 32622029 10.1016/j.ajp.2020.102190

[66] McGinty EE, Goldman HH, Pescosolido B, Barry CL. Portraying mental illness and drug addiction as treatable health conditions: Effects of a randomized experiment on stigma and discrimination. Soc Sci Med. 2015;126:73–85. 25528557 10.1016/j.socscimed.2014.12.010

[67] Taylor GM, Lindson N, Farley A, Leinberger-Jabari A, Sawyer K, Te Water Naude R, et al. Smoking cessation for improving mental health. Cochrane Database Syst Rev. 2021;3:CD013522. 33687070 10.1002/14651858.CD013522.pub2 PMC8121093

[68] Schoeler T, Monk A, Sami MB, Klamerus E, Foglia E, Brown R, et al. Continued versus discontinued cannabis use in patients with psychosis: A systematic review and meta-analysis. Lancet Psychiatry. 2016;3(3):215–25. 26777297 10.1016/S2215-0366(15)00363-6

[69] Setién-Suero E, Neergaard K, Ortiz-García de la Foz V, Suárez-Pinilla P, Martínez-García O, Crespo-Facorro B, Ayesa-Arriola R. Stopping cannabis use benefits outcome in psychosis: Findings from 10-year follow-up study in the PAFIP-cohort. Acta Psychiatr Scand. 2019;140(4):349–59. 31381129 10.1111/acps.13081

[70] Thornton LK, Baker AL, Lewin TJ, Kay-Lambkin FJ, Kavanagh D, Richmond R, et al. Reasons for substance use among people with mental disorders. Addict Behav. 2012;37(4):427–34. 22197045 10.1016/j.addbeh.2011.11.039

[71] de Haan L. It might be a wonderful opportunity when patients with a psychotic disorder use cannabis. Psychol Med. 2022;52(4):601–2. 35074041 10.1017/S0033291721003561 PMC8961333

[72] Boileau-Falardeau M, Contreras G, Gariépy G, Laprise C. Patterns and motivations of polysubstance use: A rapid review of the qualitative evidence. Health Promot Chronic Dis Prev Can. 2022;42(2):47–59. 35170930 10.24095/hpcdp.42.2.01 PMC8935897

[73] Peters EN, Schwartz RP, Wang S, O’Grady KE, Blanco C. Psychiatric, psychosocial, and physical health correlates of co-occurring cannabis use disorders and nicotine dependence. Drug Alcohol Depend. 2014;134:228–34. 24183498 10.1016/j.drugalcdep.2013.10.003 PMC3865597

