# Supplementary material for: The relationship between substance use and self-reported aspects of social functioning in patients with a psychotic disorder
Source: Eur Psychiatry. 2024 Feb 29;67(1):e21. doi: 10.1192/j.eurpsy.2024.9 (PMC10966612; doi:10.1192/j.eurpsy.2024.9)
Supplement: van der Heijden et al. supplementary material [file S0924933824000099sup001.docx]

**Supplement to:**

*The relationship between substance use and self-reported aspects of social functioning in patients with a psychotic disorder*

Sanne van der Heijden^1^, Martijn Kikkert^2^, Lieuwe de Haan^1^, Menno Segeren^3^, Simone Molman^1^, Frederike Schirmbeck^1,4*^, and Jentien Vermeulen^1*^

* Shared last author

**Affiliations:**

1. Amsterdam UMC location University of Amsterdam, Amsterdam, Netherlands
2. Department of Research, Arkin Mental Health Care, Amsterdam, The Netherlands
3. Public Health Service Amsterdam, Dept. of Healthy Living, Amsterdam, the Netherlands
4. Department of Public Mental Health, Central Institute of Mental Health, Medical Faculty Mannheim, Heidelberg University, Mannheim, Germany

**Index:**

- Supplement 1: mean scores of different self-reported aspects of social functioning per substance group in patients with a psychotic disorder
- Supplement 2: Results from linear regression model regarding the association between number of cigarettes and levels of loneliness in patients with a psychotic disorder (sensitivity analysis).
- Supplement 3: Results from linear regression model regarding the association between the amount of cannabis and social participation in patients with a psychotic disorder (sensitivity analysis).
- Supplement 4: Results from linear regression model regarding the association between differences in alcohol use and social participation in patients with a psychotic disorder (sensitivity analysis).
- Supplement 5: Results of regression models evaluating the associations between polysubstance use, no use, and self-reported aspects of social functioning in patients with a psychotic disorder (sensitivity analysis).

| Supplement 1: mean scores of different self-reported aspects of social functioning per substance group in patients with psychosis | | | | | | | | | |
| --- | --- | --- | --- | --- | --- | --- | --- | --- | --- |
|  | No smoker | Intermediate smoker | Heavy smoker | No cannabis | Non-daily cannabis | Daily cannabis | No alcohol | Non-problematic alcohol | Problematic alcohol |
| *Outcome variable* | Mean (SD) | Mean (SD) | Mean (SD) | Mean (SD) | Mean (SD) | Mean (SD) | Mean (SD) | Mean (SD) | Mean (SD) |
| Social Support (range 12-60) | 27.0 (6.7) | 26.6 (7.1) | 27.2 (6.6) | 26.8 (6.8) | 27.8 (6.4) | 27.0 (6.7) | 25.9 (6.6) | 26.9 (7.0) | 28.5 (6.4) |
| Stigmatization  Disclosure  (range 0-40)  Discrimination  (range 0-52)  Positive aspects  (range 0-20) | 18.9 (7.5)  22.5 (7.4)  8.2 (3.1) | 17.7 (6.6)  21.8 (7.6)  8.8 (2.8) | 18.7 (7.7)  25.4 (6.6)  9.1 (2.9) | 18.5 (7.4)  22.8 (7.4)  8.5 (2.8) | 17.3 (7.0)  23.7 (7.6)  8.9 (3.6) | 19.9 (6.8)  23.8 (7.0)  8.6 (3.0) | 18.9 (7.6)  23.0 (6.6)  8.3 (2.6) | 18.6 (7.0)  22.3 (7.2)  8.9 (3.0) | 17.6 (7.3)  24.3 (8.6)  8.5 (3.3) |
| Lack of social participation  (range -0.5-3.6) | 1.6 (0.7) | 1.6 (0.7) | 1.6 (0.7) | 1.6 (0.7) | 1.5 (0.6) | 1.2 (0.7) | 1.7 (0.6) | 1.6 (0.7) | 1.4 (0.7) |
| Loneliness  (range 0-6) | 2.8 (2.0) | 2.5 (1.7) | 2.7 (1.7) | 2.7 (1.9) | 2.6 (1.6) | 3.0 (1.9) | 2.8 (1.9) | 2.7 (1.9) | 2.6 (1.8) |
| Abbreviations: SD = standard deviation. Higher scores indicate poorer outcomes, with the exception of the Social Support outcome | | | | | | | | | |

| Supplement 2: Results from linear regression model regarding the association between number of cigarettes and levels of loneliness in patients with psychosis (sensitivity analysis). | | | | |
| --- | --- | --- | --- | --- |
| Variables | Estimate | SE | T | Sig. |
| (Constant) | 1.574 | 0.710 | 2.218 | 0.027 |
| Cigarettes per day | -0.017 | 0.008 | 2.045 | 0.042 |
| Age | -0.007 | 0.011 | -0.039 | 0.508 |
| Gender | -0.257 | 0.232 | -1.109 | 0.268 |
| BPRS total scale | 1.147 | 0.214 | 0.320 | <0.001 |
| Abbreviations: BPRS = Brief Psychiatric Rating Scale. SE = standard error. Higher scores indicate poorer outcomes | | | | |

| Supplement 3: Results from linear regression model regarding the association between the amount of cannabis and lack of social participation in patients with psychosis (sensitivity analysis). | | | | |
| --- | --- | --- | --- | --- |
| Variables | Estimate | SE | T | Sig. |
| (Constant) | 1.587 | 0.285 | 5.566 | <0.001 |
| Amount of cannabis (grams) | -0.092 | 0.074 | -1.247 | 0.214 |
| Age | 0.006 | 0.004 | 1.385 | 0.167 |
| Gender | -0.022 | 0.091 | -0.247 | 0.805 |
| BPRS total scale | -0.140 | 0.082 | -1.702 | 0.090 |
| Abbreviations: BPRS = Brief Psychiatric Rating Scale. SE = standard error. Higher scores indicate poorer outcomes. | | | | |

| Supplement 4: Results from linear regression model regarding the association between alcohol use and social support in patients with psychosis (sensitivity analysis). | | | | |
| --- | --- | --- | --- | --- |
| Variables | Estimate | SE | T | Sig. |
| (Constant) | 24.785 | 2.794 | 8.871 | <0.001 |
| Severe alcohol use vs. No use* | 3.394 | 1.581 | 2.147 | 0.033 |
| Age | -0.019 | 0.042 | -0.447 | 0.655 |
| Gender | 2.753 | 0.870 | 3.164 | 0.002 |
| BPRS total scale | -0.910 | 0.793 | -1.147 | 0.252 |
| Abbreviations: BPRS = Brief Psychiatric Rating Scale. SE = standard error. *No use is the reference category. AUDIT threshold was set at 16 or higher, indicating severe problematic alcohol use. | | | | |

| Supplement 5: Results of regression models evaluating the associations between polysubstance use, no substance use, and different self-reported aspects of social functioning | |
| --- | --- |
|  | *Polysubstance use vs. No substance use^^^* |
| *Outcome variable* | Estimate (SE) [95% CI], p-value |
| Social Support | 1.271 (1.103) [-0.902 – 3.443], p = 0.251 |
| Stigmatization - Disclosure  Stigmatization - Discrimination  Stigmatization - Positive aspects | -0.818 (1.348) [-3.476 – 1.839], p = 0.544  0.596 (1.318) [-2.003 – 3.195], p = 0.651  1.092 (0.562) [-0.016 – 2.199],p = 0.053 |
| Lack of social participation | -0.058 (0.111) [ -0.277 – 0.161], p = 0.602 |
| Loneliness | -0.569 (0.287) [-1.135 – -0.003], **p = 0.049** |
| Estimate (Standard Error). [95% CI] = 95% Confidence Interval. Higher scores indicate poorer outcomes, with the exception of the Social Support outcome. ^^^No use is the reference category against which polysubstance use is compared. All estimates are corrected for age, gender, and psychopathology. | |
